# Family history of cardiovascular disease and risk of premature coronary heart disease: A matched case-control study

**DOI:** 10.12688/wellcomeopenres.15829.2

**Published:** 2020-06-12

**Authors:** Manas Chacko, P. Sankara Sarma, Sivadasanpillai Harikrishnan, Geevar Zachariah, Panniyammakal Jeemon

**Affiliations:** 1 Achutha Menon Centre for Health Science Studies, Sree Chitra Tirunal Institute for Medical Sciences and Technology, Trivandrum, Kerala, 695011, India; 2 Department of Cardiology, Sree Chitra Tirunal Institute for Medical Sciences and Technology, Trivandrum, Kerala, 695011, India; 3 Department of Cardiology, Mother Heart Care, Mother Hospital, Thrissur, Kerala, India

**Keywords:** Cardiovascular disease, premature coronary heart disease, family history, India

## Abstract

**Background: **Self-reported family history of cardiovascular disease (CVD) is an independent risk factor for future coronary heart disease (CHD) events. However, inclusion of family history of CVD in the traditional risk scores failed to improve risk prediction of CHD. It is proposed that family history of CVD may substantially increase the risk of CHD among younger individuals.

**Methods: **We conducted a matched case-control study with 170 hospital-based premature CHD patients (<55 years in men and <65 years in women) from a tertiary care centre in Thiruvananthapuram, Kerala and age and sex matched community-based controls in 1:1 ratio. Conditional logistic regression analysis was conducted to assess the independent association of family history of cardiovascular disease (CVD) and premature CHD. We estimated McNemar's odds ratios and their 95 percent confidence intervals.

**Results: **The prevalence of any family history of CVD and CHD in the control population was 24% and 21%, respectively. The family history of CVD was independently associated with premature CHD (odds ratio (OR) = 9.0; 95% confidence interval (CI) 4.7–17.3). There was a dose-response relationship between family history and premature CHD as the risk increased linearly with increase in number of affected family members.

**Conclusions: **Family history of CVD is an independent risk factor for premature CHD. The risk of premature CHD increases linearly with increase in number of affected family members. Collecting family history beyond parental history of CVD is important for risk stratification. Targeting young individuals with family history of CVD for intensive risk reduction interventions may help to prevent future events.

## Introduction

Globally, cardiovascular diseases (CVD) remain one of the single largest contributors to mortality ^1^. The CVD epidemic is advancing rapidly in low- and middle-income country (LMIC) settings and India is not an exception ^2^. Coronary heart diseases (CHD) is a major constituent of CVD in India ^3^, which is attributable to approximately two-thirds of the total CVD burden. In India, CHD affects in the productive age groups and younger people are affected disproportionately as compared to high-income country settings ^4^.

Family history of CVD is an important risk factor for development of future CHD ^5^. However, inclusion of family history of CVD in the traditional risk scores failed to improve risk prediction of CHD ^6^. It has been however postulated that family history is strongly associated with development of premature CHD events. Only a few risk equations, like QRISK2 ^7^, JBS3 ^8^ and Reynolds ^9^, use family history of CVD for assessing future risk. Since the risk scores consider age as an important risk factor for CHD, the risk associated with family history in premature CHD gets diluted in the risk equations. Hence, it is important to study the significance of a positive family history of CVD in a subgroup of patients with premature CHD.

Family history is an important constituent of the health history of any patient and may imply the shared family behaviour, environment and genetic heritage. Although a detailed family history that includes number of relatives, age, and sex of the affected individual may make it relatively harder to acquire during clinical visits, the complexity in detailed family history collection is similar to other behavioural risk measurements. Further, the role of detailed family history in premature CHD is not studied in detail in the LMIC settings due to the undervaluation of such data collection efforts.

Ideally, CVD prevention should start early in life for limiting the cumulative lifetime exposure to risk conditions and to achieve global risk reduction. However, it would be difficult to target everyone in the CHD prevention models or programme. Risk stratification based on a relatively easy and cheap measurement tools may help to identify high risk sub-groups for intensive CVD risk reduction. Family history of CVD often demonstrates lifetime exposure to shared family behaviour and environment ^10^. Although family history is a non-modifiable risk factor, it is possible to reduce the total CHD risk among individuals with a strong family history of CHD by modifying their exposure to other known risk factors early in life ^11^. We conducted a study to assess the strength of independent relationship between detailed family history of CVD and premature CHD among Indians.

## Methods

### Study design

We conducted a matched case-control study in Kerala, India.

### Study settings

The cases were identified from a tertiary care speciality hospital for cardiovascular conditions in Thiruvananthapuram district, Kerala, India. The controls were identified from a representative community based prevalence survey of CHD and their risk factors from Thiruvananthapuram district, Kerala ^12^. The Cardiological Society of India Kerala chapter Coronary artery disease and its Risk factors Prevalence (CSI Kerala CRP) study was a cross-sectional survey to assess the prevalence of coronary artery disease and its risk factors in Kerala. The CSI Kerala CRP study collected data from both urban and rural areas of three different districts in Kerala. We used the data collected from Thiruvananthapuram district to identify the controls. The data collection methods of CSI Kerala CRP study have been explained in detail elsewhere ^13, 14^.

### Study population

Patients with established CHD either confirmed by coronary angiogram (at least single vessel disease with more than or equal to 70% disease) or evident myocardial infarction from treatment history were selected as cases. We restricted the CHD cases to men less than 55 years of old and women less than 65 years of old (premature CHD) ^15, 16^ and included cases only from Thiruvananthapuram district, Kerala, India. Eligible cases reported to a tertiary care speciality hospital during the period of 01/09/2015 to 31/08/2017 were included in the study. We used the electronic patient records to identify the cases based on the eligibility criteria.

Healthy people with no history of cardiovascular disease and no signs and symptoms of CHD by electrocardiogram (ECG) and Rose Angina Questionnaire (RAQ) ^17, 18^ were considered as controls. The controls were identified from a representative cross-sectional survey conducted in Thiruvananthapuram district, Kerala, India as part of the CSI-CRP study ^12^. Individuals with any previous history of cardiovascular disease, chronic lung disease, and cancer were excluded from the study as controls. We also excluded pregnant women or people with a severe form of disability.

### Study variables and data collection

The key exposure variable of interest was family history of CVD. We defined the family history of CVD as a history of CHD or stroke among any first-degree relatives of the study participants. Additionally, we collected information on the number of first-degree relatives affected, their age at the time of diagnosis and sex ^16^. However, we did not capture data on twins and consanguinity as part of family history assessment. We used data derived from detailed assessment of behavioural risk factors of CVD using a structured tool. Additionally, we measured height and weight of all cases and utilised the already measured data on these variables from controls. Past history of diabetes, hypertension and dyslipidaemia was obtained. A structured interview schedule, which was translated in Malayalam language, was used for data collection from cases and controls (see *Extended data* ^19^). Further, we obtained fasting glucose and blood pressure data from both cases and controls. The data collection tools and procedures were exactly same in cases and controls. We followed standard techniques according to the WHO STEPS manual ^20^.

### Definitions

Family history of CHD or stroke in any first-degree relatives was defined as ‘any family history’ of CVD. Additionally, we used age at diagnosis criteria (<55 years in men and <65 years in women) to define the family history of premature CVD. Father, mother, brothers and sisters were defined as first-degree relatives. Stepfathers, stepmothers, stepbrothers and stepsisters were excluded. Those who ever used any form of tobacco were defined as tobacco users. Those who ever used alcohol were defined as alcohol users. Moderate to vigorous physical activity of less than 150 minutes per week was defined as sedentary lifestyle. People who were on treatment for hypertension or having systolic blood pressure more than or equal to 140 mm of Hg, or diastolic blood pressure more than or equal to 90 mm of Hg were defined as individuals with hypertension. Diabetes mellitus was defined as previous history of treatment for high blood glucose or fasting blood glucose more than or equal to 126 mg/dl. Treatment history for hyperlipidaemia was considered as past medical history of dyslipidaemia. Body mass index higher than 25 kg/m ^2^ was defined as overweight.

### Sample size

The sample size was calculated with STATA version 13 ^21^ and as per the method described by Dupont ^22, 23^. The sample size was calculated with a power of 80% and two-sided confidence level of 95%. The expected prevalence of family history of CVD among control group was considered as 21% ^12^. We calculated the sample size to detect a minimum odds ratio (OR) of 2.4 as described in several other studies ^24– 28^. For a matched case-control study at a case to control ratio of 1:1, the required sample size was 162 pairs of cases and controls. We further rounded the sample size to 170 cases and 170 matched controls.

### Ethical issues

The Institutional Ethics Committee (IEC) of Sree Chitra Tirunal Institute for Medical Sciences, Trivandrum approved the study (IEC Approval letter: SCT/IEC/1044/MAY-2017). The interview for data collection and all measurements were conducted after obtaining a written informed consent from each study participant. Privacy was ensured during the time of interview and confidentiality of all the information collected was maintained. The participants had the freedom to refuse participation at the beginning or during any stage of data collection.

### Study database and matching

A data entry platform was created using EpiData Manager Version 4.2. We used EpiData Entry Client Version 4.2 ^29^ for data entry in cases and exported the data set as .csv files. For preparing dataset of controls, we excluded all probable, possible and definite cases of coronary heart disease based on the ECG criteria and Rose Angina Questionnaire from CSI-Kerala CRP study dataset. We then conducted an exact matching of cases and controls based on age and gender with a ratio of 1:1 using IBM SPSS Statistics for Windows with Python Essentials version 25 using fuzzy command ^30^. The final analysis was conducted in a perfectly matched set of 170 cases and controls.

### Data analysis

We performed all data analysis in STATA Version 13 ^21^. Continuous variables were presented as mean and standard deviation. Categorical variables were presented as frequency and percentage. We performed conditional logistic regression and estimated McNemar’s odds ratio with 95% confidence interval (CI). All exposure variables that were associated with CHD outcome in the bivariate analysis and known risk factors were taken up for multivariate analysis.

## Results

### Descriptive data in the study population

There were 170 cases and 170 paired controls in the study. The proportion of women in the study was 25% in each group. The mean age of the study population was 49±7 years. A small proportion (2.6%) of the study population was illiterate. The median years of formal education was 10 with an interquartile range from 7 to 12 years. Near to one-third of the study population (31%) held a BPL ration card (low socio-economic group with access to free or subsidised food items). The prevalence of tobacco use in men was 55%. Half (50%) of men were ever users of alcohol. Nearly half (44%) of the study population followed sedentary lifestyle. The proportion of diabetes and hypertension in the study population was 39% and 41%, respectively. A small proportion of the study population was on treatment for dyslipidaemia (6.5%). Individual-level results for each participant in each group are available as *Underlying data* ^31^.

### Distribution of confounding variables in cases and controls

Due to perfect matching, the mean age and proportion of men in cases and controls were the same ( Table 1). More than one-third of both cases and controls reported below 10 ^th^ standard education (37.1% and 38.2%, p=0.99). More than one-third of the cases (37.1%) belonged to low socio-economic group, while it was one-fourth (25.3%) in controls. Tobacco and alcohol use were similar in cases and controls. The overweight proportion was also similar in cases and controls. Almost half of cases reported hypertension, while it was less than one-third (31.2%) in controls (p<0.001). Similarly, diabetes proportion was higher in cases as compared to controls (48.2% vs 29.4%, p=0.001). The proportion of participants with dyslipidaemia was also higher in cases as compared to controls (9.4% vs 3.5%, p=0.05).

**Table 1.** General characteristics of the study population.

Variables	Cases n=170	Controls n=170	P-value	Crude OR (95% CI)
Age in years, mean (SD)	48.6 (7.2)	48.6 (7.2)	0.999	-
Men, n (%)	127 (74.7)	127 (74.7)	0.999	-
Below 10 ^th^ standard of education, n (%)	64 (37.6)	65 (38.2)	0.999	1.0 (0.6 - 1.6)
Below poverty line, n (%)	63 (37.1)	43 (25.3)	0.026	1.9 (1.1 - 3.1)
Tobacco use, n (%)	80 (47.1)	65 (38.2)	0.125	1.7 (1.0 - 2.9)
Alcohol use, n (%)	68 (40.0)	59 (34.7)	0.370	1.3 (0.8 - 2.2)
Sedentary lifestyle, n (%)	81 (47.6)	68 (40.0)	0.190	1.4 (0.9 - 2.2)
Overweight, n (%)	76 (44.7)	77 (45.3)	0.999	1.0 (0.6 - 1.5)
Hypertension, n (%)	86 (50.6)	53 (31.2)	<0.001	2.6 (1.6 - 4.3)
Diabetes, n (%)	82 (48.2)	50 (29.4)	0.001	2.3 (1.4 - 3.6)
Hyperlipidaemia, n (%)	16 (9.4)	6 (3.5)	0.045	3.0 (1.1 - 8.3)

OR, odds ratio; CI, confidence interval.

### Clinical characteristics of cases

More than half of cases (56.5%) had ST-elevation myocardial infarction (STEMI). The proportion of STEMI was disproportionately higher in men as compared to women (61.4% vs 41.9%: Table 2). Triple vessel disease was diagnosed in nearly one-third of cases based on angiogram (30.6%). Left main coronary artery disease was present in 4.1% of cases.

**Table 2.** Mode of presentation and angiographic profile of the cases.

Variables (Cases)	Male n=127	Female n=43	Total n=170
Mode of presentation, n (%)			
STEMI	78 (61.4)	18 (41.9)	96 (56.5)
Non-STEMI	13 (10.2)	6 (14.0)	19 (11.2)
USA	7 (5.5)	5 (11.6)	12 (7.1)
AOE	26 (20.5)	12 (27.9)	38 (22.4)
DOE	3 (2.4)	2 (4.7)	5 (2.9)
Coronary artery disease severity, n (%)			
Single vessel disease	32 (25.2)	17 (39.5)	49 (28.8)
Double vessel disease	51 (40.2)	10 (23.3)	61 (35.9)
Triple vessel disease	38 (29.9)	14 (32.6)	52 (30.6)
No or minor disease	6 (4.7)	2 (4.7)	8 (4.7)
LMCA disease, n (%)	5 (3.9)	2 (4.7)	7 (4.1)

STEMI, ST elevated myocardial infarction; USA, unstable angina; AOE, angina on exertion; DOE, dyspnoea on exertion; LMCA, left main coronary artery disease.

### Family history exposure status in cases and controls

**Figure 1.**
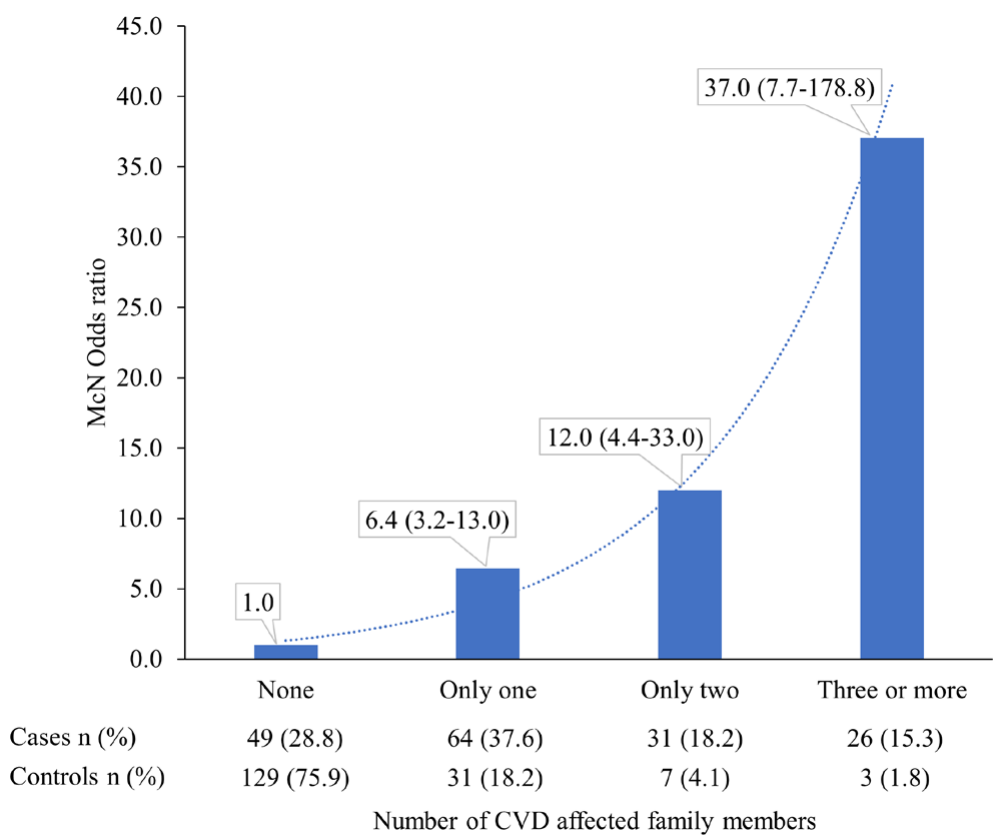
Difference in the association of number of cardiovascular disease (CVD)-affected family members with premature CHD. Data shown are odds ratios (95% confidence intervals).

Any family history of CVD (CHD and stroke combined) was reported in 24.1% of controls, while it was 71.2% in cases (p<0.001). Similarly, proportion of participants with any family history of CHD was substantially lower in controls (20.6%) as compared to cases (65.3%). Family history of premature CVD was high among cases (48.8%) as compared to controls (11.2%). Substantially higher proportion of cases reported parental family history of both CVD and CHD as compared to their matched controls ( Table 3). Substantially higher proportion of cases as compared to controls reported only one, two and more than two affected family members (37.6% vs 18.2%, 18.2% vs 4.1%, and 15.3% vs 1.8%, respectively; Figure 1).

**Table 3.** Strength of association of family history and premature coronary heart disease (CHD).

Variables	Cases n=170	Controls n=170	P-value	Crude OR (95% CI)
Any family history of CVD, n (%)	121 (71.2)	41 (24.1)	<0.001	9.0 (4.7 – 17.3)
Parental family history of CVD, n (%)	104 (61.2)	36 (21.2)	<0.001	7.2 (3.8 – 13.5)
Siblings history of CVD, n (%)	56 (32.9)	8 (4.7)	<0.001	9.0 (3.9 - 20.9)
Any family history of CHD, n (%)	111 (65.3)	35 (20.6)	<0.001	7.9 (4.2 – 14.8)
Parental family history of CHD, n (%)	91 (53.5)	31 (18.2)	<0.001	5.6 (3.1 – 10.1)
Siblings history of CHD, n (%)	49 (28.8)	7 (4.1)	<0.001	9.4 (3.7 - 23.6)
Any family history of premature CVD, n (%)	83 (48.8)	19 (11.2)	<0.001	7.4 (3.8 - 14.3)
Parental family history of premature CVD, n (%)	52 (30.6)	14 (8.2)	<0.001	5.7 (2.7 - 12.2)
Siblings history of premature CVD, n (%)	46 (27.1)	5 (2.9)	<0.001	11.2 (4.0 - 31.3)
Any family history of premature CHD, n (%)	73 (42.9)	18 (10.6)	<0.001	7.1 (3.5 - 14.3)
Parental family history of premature CHD, n (%)	43 (25.3)	14 (8.2)	<0.001	4.6 (2.2 - 9.9)
Siblings history of premature CHD, n (%)	41 (24.1)	4 (2.4)	<0.001	13.3 (4.1 - 43.1)

OR, odds ratio; CI, confidence interval; CVD, cardiovascular disease.

### Family history and the risk of premature coronary heart disease

Both parental and ‘any family history’ of CVD was associated with premature CHD (OR=7.2; 95%CI: 3.8-13.5 and OR=9.0; 95% CI: 4.7-17.3, respectively) in the unadjusted models. Family history of premature CVD was also strongly associated with premature CHD (OR=7.4; 95% CI: 3.8 - 14.3). Adjustment for other potential confounders did not attenuate the odds ratio ( Table 4). In the multivariate model, other major risk factors of premature CHD were low socio-economic status, tobacco use, sedentary lifestyle, hypertension, diabetes and hyperlipidaemia ( Table 4). The number of affected individuals in the family history of CVD showed a dose-response relationship with premature CHD ( Figure 1). The OR associated with only one, two or more than two affected family members and premature CHD were 6.4 (95%CI: 3.2-13.0), 12.0 (95%CI: 4.4-33.0) and 37.0 (95%CI: 7.7-178.8), respectively.

**Table 4.** Independent association of family history of cardiovascular disease (CVD) with premature coronary heart disease.

1:1 matched 170 pairs	Model 1 Adjusted OR (95% CI)	Model 2 Adjusted OR (95% CI)	Model 3 Adjusted OR (95% CI)	Model 4 Adjusted OR (95% CI)
Below 10 ^th^ std of education	0.7 (0.3 - 1.4)	0.7 (0.4 - 1.5)	0.6 (0.3 - 1.2)	0.7 (0.4 - 1.4)
Below poverty line	2.4 (1.0 - 5.6)	2.3 (1.1 - 5.1)	2.6 (1.1 - 5.9)	2.3 (1.1 - 4.8)
Tobacco use	4.9 (1.7 - 14.1)	3.6 (1.5 – 9.0)	4.2 (1.6 - 11.4)	3.4 (1.4 - 8.1)
Alcohol use	0.6 (0.3 - 1.3)	0.7 (0.4 - 1.6)	0.6 (0.3 - 1.3)	0.7 (0.3 - 1.4)
Sedentary lifestyle	2.2 (1.0 - 4.7)	2.0 (1.0 - 4.1)	1.9 (0.9 - 3.9)	1.8 (0.9 - 3.6)
Overweight	1.1 (0.5 - 2.1)	1.1 (0.6 - 2.1)	1.0 (0.5 – 2.0)	1.1 (0.6 - 1.9)
Hypertension	4.4 (1.9 – 10.0)	3.7 (1.7 - 7.8)	4.2 (1.9 - 9.3)	3.6 (1.8 - 7.3)
Diabetes	2.3 (1.2 - 4.8)	2.0 (1.0 - 3.8)	2.2 (1.1 - 4.3)	2.0 (1.1 - 3.7)
Hyperlipidaemia	5.0 (1.1 - 21.5)	3.8 (1.0 - 14.1)	4.9 (1.2 - 20.0)	3.2 (1.0 - 11)
Any FH of CVD	14.9 (6.1 – 36.0)	-	-	-
Parental FH of CVD	-	9.5 (4.3 - 20.9)	-	-
Any FH of CHD	-	-	12.4 (5.4 - 28.5)	-
Parental FH of CHD	-	-	-	7.1 (3.4 - 14.6)
Any FH of premature CVD *	8.9 (4.0 - 19.8)	-	-	-
Parental FH of premature CVD *	-	6.6 (2.8 - 15.8)	-	-
Any FH of premature CHD *	-	-	8.3 (3.6 - 19.1)	-
Parental FH of premature CHD *	-	-	-	5.1 (2.1 - 12.4)

OR, odds ratio; CI, confidence interval; CHD, coronary heart disease; FH, Family History. * All are separate models adjusted for the same set of variables as in Model 1, Model 2, Model 3 and Model 4, respectively.

## Discussion

We demonstrate that independent of age, sex, and other major risk factors, family history of CVD is strongly associated with premature CHD. The strong association with large effect size and dose response relationship of number of family members affected and premature CHD clearly indicate a potential causal relationship. The exact matching for age and sex probably helped us to measure the association totally independent of these two key risk factors of CHD.

The dose response relationship observed in our study with the number of CVD affected family members and premature CHD is consistent with findings from the INTERHEART study ^32^. Further, family history not only increases the risk of disease but also increases the severity of the disease ^33^. However, the exposure variable in the INTERHEART study was just limited to parental history of acute myocardial infarction (AMI) ^32^. Only 12% of the control population reported parental history of myocardial infarction in the INTEHEART study, while any family history of CVD was reported in almost a quarter of the control population in our study. Further, the controls in our study were selected from a representative cross-sectional survey in the general population and it was conducted in the same geographical location from where we have drawn the cases for the study. We clearly demonstrate that individuals with any family history of CVD, which consist of nearly a quarter of the population, is clearly a high risk group to target for early interventions to prevent premature CHD.

We restricted our cases to premature CHD before the age of 55 years in men and 65 years in women. More than two third of cases in our study reported a positive family history. Importantly, one third of them reported two or more affected family members. This is far higher than the proportion of AMI cases with parental history of AMI in the INTERHEART study ^32^. The higher proportion of affected individuals is largely due to restriction of cases to premature CHD in our study. Similar findings are reported in other studies from India. For example, a registry of young CHD patients from India showed similar higher prevalence of family history ^34^. Additionally, in a very large community-based study from the USA, the reported prevalence of positive family history of CHD was 72% among patients with premature coronary artery disease ^35^.

The failure of family history to improve risk prediction beyond traditional risk factors in standard risk equations should not be construed as proof for no true association of family history with CHD. Our data clearly demonstrate that once you balance the risk due to age and sex, family history is indeed a strong predictor of premature CHD. Therefore, targeted intervention approaches in people with family history of CVD may help in the prevention or delay of CHD in the productive year of life. Although part of the risk associated with family history is mediated through genetic mechanisms, it is worthwhile to note that lifestyle interventions and choices are equally or more effective in individuals with high genetic risk for development of CHD ^36^. Given that even genetic risk gets attenuated by favourable lifestyle, the strategy to target individuals with family history of CHD to mitigate both the genetic and behavioural risk early in life is appropriate and essential for prevention of premature events. However, screening of the high-risk families and targeted risk reduction strategies are often ignored in primary care settings even in developed countries ^37^. We need to develop public health policies, which support targeted lifestyle intervention in individuals with family history of CHD. The programme of lifestyle intervention in individuals with family history of premature CHD (PROLIFIC trial ^38^) shows that such interventions are acceptable, desirable and feasible in LMIC settings ^39^. Detailed results of the PROLIFIC trial ^38^ will provide further evidence to advocate for family based strategies in cardiovascular risk reduction and their likely impact at the societal level.

## Strengths and limitations

The perfect matching for age and sex in our case control study and adoption of conditional logistic regression for estimation of effect sizes minimised bias due to confounding. The representativeness of cases and controls improved both the internal and external generalisability of the findings. Since we selected only survived cases, our study is subjected to survival bias. However, family history of CHD is reported more among severe cases of CHD as compared to less severe cases. Hence, the survival bias may only dilute the true effect size associated with family history of CHD. Additionally, as in any other case-control study, our study is also subjected to recall bias. However, the use of standardised measurement tools and adequate explanation of the purpose of the study to both cases and controls would have minimised the recall bias in our study. Selection of controls from a four-year old survey may have further influenced our results. Finally, referral bias would have influenced the study finding as the cases were identified only from one tertiary care super speciality hospital.

## Conclusion

The family history of cardiovascular disease is associated with premature coronary artery disease. The CHD risk increases linearly with increase in number of affected family members. Individuals with family history of CVD should be targeted for cardiovascular risk reduction interventions. Counselling centres in hospitals for the immediate relatives of patients with CVD may be an attractive policy option with likely public health impact.

## Data availability

### Underlying data

Figshare: Family history of cardiovascular disease and risk of premature coronary heart disease: A matched case-control study. https://doi.org/10.6084/m9.figshare.12058230.v4 ^31^.

This project contains the raw individual-level data for each participant in CSV, DAT and DTA formats.

### Extended data

Figshare: Family history of cardiovascular disease and risk of premature coronary heart disease: A matched case-control study. https://doi.org/10.6084/m9.figshare.12066624.v2 ^19^.

This project contains the data collection tool used in this study.

Data are available under the terms of the Creative Commons Attribution 4.0 International license (CC-BY 4.0).
