# Supplementary material for: Family history of cardiovascular disease and risk of premature coronary heart disease: A matched case-control study
Source: Wellcome Open Res. 2020 Jun 12;5:70. Originally published 2020 Apr 16. [Version 2] doi: 10.12688/wellcomeopenres.15829.2 (PMC7256470; doi:10.12688/wellcomeopenres.15829.2)
Supplement: Peer review history

# Peer review: Reviewer response for version 1

**DOI:** 10.21956/wellcomeopenres.17363.r38472

**Published:** 2020-05-28

**Authors:** Amit Malviya

**Affiliations:** 1 Department of Cardiology, Northeastern Indira Gandhi Regional Institute of Health and Medical Sciences (NEIGRIHMS), Shillong, Meghalaya, India

In this article Manas Chacko *et al.* report a strong association of family history, specifically in first degree relatives, with incidence of premature coronary artery disease in a matched case-control study with 170 hospital-based premature CHD patients from south India. On technical grounds and organization of the manuscript, the study holds strong. The authors have extensively justified the relevance of study. Overall a very important topic with a well conducted and well written study. However, being a case control study, the results are subjected to various confounding factors and bias (Tenny and Hoffman, 2020 ^1^). Many patients may not be able to recall the family history correctly especially if it was not severe enough. The data may not be reflective of the general population as cases were picked up from a tertiary hospital setting and controls were selected from old registry data. I congratulate the authors and recommend for indexing.

Is the work clearly and accurately presented and does it cite the current literature?

Yes

If applicable, is the statistical analysis and its interpretation appropriate?

Yes

Are all the source data underlying the results available to ensure full reproducibility?

Partly

Is the study design appropriate and is the work technically sound?

Yes

Are the conclusions drawn adequately supported by the results?

Yes

Are sufficient details of methods and analysis provided to allow replication by others?

Yes

Reviewer Expertise:

Rheumatic heart disease

I confirm that I have read this submission and believe that I have an appropriate level of expertise to confirm that it is of an acceptable scientific standard.

## References

[1] : Case Control Studies. 28846237

# Response

**Published:** 2020-06-09

**Authors:** Panniyammakal Jeemon

**Affiliations:** Sree Chitra Tirunal Institute for Medical Sciences and Technology, Trivandrum, India

Thank you very much for the positive feedback and comments. 

We agree that confounding and recall bias are major issues in case-control study. We have acknowledged them as limitations in the paper.

# Peer review: Reviewer response for version 1

**DOI:** 10.21956/wellcomeopenres.17363.r38575

**Published:** 2020-05-20

**Authors:** Nitin Kapoor, Vineeth Thomas

**Affiliations:** 1 Department of Endocrinology, Christian Medical College, Vellore, Tamil Nadu, India; 2 Melbourne school of population and global health, University of Melbourne, Melbourne, Victoria, Australia; 3 Department of Medicine, Christian Medical College, Vellore, India

This study highlights the importance of assessing family history in predicting the risk of cardiovascular disease. The authors found that there is a strong and independent association of family history with premature CHD which increased linearly with the increase in number of affected first degree relatives.

 The introduction is well written and comprehensive. It justifies the need for this study.

 Would suggest adding the following points that may be important in the Indian context:

1. The role of consanguinity is another important aspect while determining the effect of family history on chronic disorders (Kapoor *et al.*, 2019 ^1^). This is especially important as India and some other neighboring lower-middle income countries harbor some of the world’s largest consanguineous populations (Hamamy, 2012 ^2^).
2. Though we agree with the authors that the mean age of many non-communicable diseases is lower in the Indian setting when compared to western populations, while making these comparisons it is important to also cite that the mean of the average Indian population and the life expectancy is also much lower than these western populations. Hence it is expected that the mean age of these disease cohorts will also be lower in India as there aren’t many people in the eight and ninth decade to shift the mean to the right.

The methodology section is also very well explained. Would suggest the following points for modification:

1. Would suggest modifying: Page 3, column 2, line 10 “were explained” – “have been explained”.
2. Study variables – It may be good to mention if while capturing family history, data on twins, consanguinity, unexplained death of any of the first-degree relatives was also captured. 
3. It is good to note that history of diabetes, hypertension and dyslipidemia was captured from the first-degree relatives. But would be good to mention if additional data on other risk factors including smoking, alcohol consumption etc. was also captured. As these may also influence the occurrence of premature CVD in these relatives. 
4. It has been mentioned that stepbrothers and stepsisters were excluded. But I presume stepmothers and stepfathers were also excluded, if so may mention all or remove the line on brothers and sisters.
5. For diagnosis of diabetes – may be preferable and scientifically more apt to use the term “glucose” instead of “sugar”.
6. For the definition of overweight, it may be advisable to use the WHO Asia-Pacific cutoff to define overweight. Currently the western cutoff has been used.
7. For sample size calculation, the prevalence in the quoted study is incorrectly stated. In the paper by Krishnan *et al.* the actual prevalence of family history of CVD is 18% unlike 21% that has been cited and used for sample size calculation in this manuscript (Krishnan *et al.*, 2016 ^3^).
8. Statistics in this manuscript are clearly explained.

The results and discussion section is very well explained. Good comparisons to the INTERHEART study have been made. However, in the discussion, a bit more expansion of the different proposed patho-physiological mechanisms on how affected family members may lead to higher risk in their progeny could be added. Mention of factors right from mother’s metabolic health during pregnancy, birth weight, common dietary practices etc. may further be highlighted in the discussion. 

 Overall, I think this is an excellent study, meets the standards of the journal and carries a very important public health message to stimulate further prospective research to study the impact of an early intervention in reducing cardiometabolic diseases in individuals with a strong family history of CVD.

Is the work clearly and accurately presented and does it cite the current literature?

Yes

If applicable, is the statistical analysis and its interpretation appropriate?

Yes

Are all the source data underlying the results available to ensure full reproducibility?

Yes

Is the study design appropriate and is the work technically sound?

Yes

Are the conclusions drawn adequately supported by the results?

Yes

Are sufficient details of methods and analysis provided to allow replication by others?

Yes

Reviewer Expertise:

Obesity and metabolic syndrome ; Osteoporosis and Pituitary disorders

We confirm that we have read this submission and believe that we have an appropriate level of expertise to confirm that it is of an acceptable scientific standard.

## References

[1] : Genetics of obesity in consanguineous populations - A road map to provide novel insights in the molecular basis and management of obesity. *EBioMedicine*.2019;40: 10.1016/j.ebiom.2019.01.004 33-34 10.1016/j.ebiom.2019.01.004 30638866PMC6412867

[2] : Consanguineous marriages : Preconception consultation in primary health care settings. *J Community Genet*.2012;3(3) : 10.1007/s12687-011-0072-y 185-92 10.1007/s12687-011-0072-y 22109912PMC3419292

[3] : Prevalence of coronary artery disease and its risk factors in Kerala, South India: a community-based cross-sectional study. *BMC Cardiovasc Disord*.2016;16: 10.1186/s12872-016-0189-3 12 10.1186/s12872-016-0189-3 26769341PMC4712491

# Response

**Published:** 2020-06-09

**Authors:** Panniyammakal Jeemon

**Affiliations:** Sree Chitra Tirunal Institute for Medical Sciences and Technology, Trivandrum, India

Thank you very much for your positive comments.

We did not capture data on twins and consanguinity as part of family history assessment. It was also not part of the questionnaire that we used in the assessment of family history in the control population. 

We agree that the mean age of the Indian population is lower than that in the high-income countries. However, disproportionately higher number of affected individuals in the productive life years in India has important consequences in terms of productivity loss, and economic burden. We just wanted to highlight this point in the introduction section. 

Sudden cardiac death of the first-degree relatives was captured. However, the unexplained death of first-degree relatives was not considered as a positive family history.

We have only included first degree blood relatives in the family history assessment. We have excluded stepfathers, stepmothers, stepbrothers and stepsisters. 

The definition of of overweight did not show any impact on the relationship between family history and CVD. 

In the paper by Krishnan *et al.* the age-adjusted prevalence of family history was 18%. We had access to the whole data and we calculated the un-adjusted prevalence of family history of CVD in Trivandrum. The un-adjusted prevalence (21%) was used in the estimation of sample size. 

We have already discussed the role of both genetics and environment in the pathway to premature coronary heart disease. Further, we have highlighted the importance of lifestyle choices in individual with higher genetic risk. We agree that mother's metabolic health during pregnancy and birth weight are also important determinants of future CVD risk. 

Once again, thank you very much for the comments and critique on our article.
